# Genetic association of FMRP targets with psychiatric disorders

**DOI:** 10.1038/s41380-020-00912-2

**Published:** 2020-10-19

**Authors:** Nicholas E. Clifton, Elliott Rees, Peter A. Holmans, Antonio F. Pardiñas, Janet C. Harwood, Arianna Di Florio, George Kirov, James T. R. Walters, Michael C. O’Donovan, Michael J. Owen, Jeremy Hall, Andrew J. Pocklington

**Affiliations:** 1 Neuroscience and Mental Health Research Institute, Cardiff University, Cardiff, UK; 2 MRC Centre for Neuropsychiatric Genetics and Genomics, Division of Psychological Medicine and Clinical Neurosciences, Cardiff University, Cardiff, UK

**Keywords:** Neuroscience, Genetics, Schizophrenia, Bipolar disorder, Depression

## Abstract

Genes encoding the mRNA targets of fragile X mental retardation protein (FMRP) are enriched for genetic association with psychiatric disorders. However, many FMRP targets possess functions that are themselves genetically associated with psychiatric disorders, including synaptic transmission and plasticity, making it unclear whether the genetic risk is truly related to binding by FMRP or is alternatively mediated by the sampling of genes better characterised by another trait or functional annotation. Using published common variant, rare coding variant and copy number variant data, we examined the relationship between FMRP binding and genetic association with schizophrenia, major depressive disorder and bipolar disorder. High-confidence targets of FMRP, derived from studies of multiple tissue types, were enriched for common schizophrenia risk alleles, as well as rare loss-of-function and de novo nonsynonymous variants in schizophrenia cases. Similarly, through common variation, FMRP targets were associated with major depressive disorder, and we present novel evidence of association with bipolar disorder. These relationships could not be explained by other functional annotations known to be associated with psychiatric disorders, including those related to synaptic structure and function. This study reinforces the evidence that targeting by FMRP captures a subpopulation of genes enriched for genetic association with a range of psychiatric disorders.

## Introduction

Fragile X mental retardation protein (FMRP) binds selected mRNA species to repress their translation [1–5]. In the brain, FMRP is highly, and dynamically, expressed in neurons, where it regulates the dendritic synthesis of proteins [6, 7], many of which are modulators of synaptic plasticity [1]. The loss of FMRP function causes fragile X syndrome [8], characterised by abnormal dendritic morphology, impaired learning and memory, autism and a high prevalence of seizures [9].

The mRNA targets of FMRP have received additional attention due to their enrichment for genes harbouring risk to psychiatric disorders. A set of 842 high-confidence FMRP targets, originating from a study by Darnell et al. [1], have been reported to be enriched for genetic association with schizophrenia [10–17], autism [18–21] and major depressive disorder [22]. In the case of schizophrenia, not only is this association robust across genome-wide association studies, but it is also seen in studies of rare variants that confer risk for the disorder [10–16].

Whilst the case for the involvement of some FMRP targets in psychiatric disorders is unequivocal, FMRP targets represent long, brain-expressed transcripts [23] with considerable overlap with other sets of genes enriched for genetic association with psychiatric disorders, including those encoding synaptic proteins [1, 24]. This has led to speculation that the association between psychiatric disorders and FMRP targets is driven not by the property of being targets of FMRP per se, rather that it reflects association to one or more functional sets of genes that also happen to be overrepresented in the FMRP target set [23]. Furthermore, FMRP targets were defined by applying a cut-off to a probabilistic scale of FMRP binding [1], though the relationship between these binding statistics and genetic association with psychiatric disorders has not been investigated.

In the present study, we aimed to: (1) establish whether the association of FMRP target genes with schizophrenia correlates with binding confidence; (2) determine whether the FMRP gene set association can be explained by alternative characterisation or functional annotation of genes; and (3) demonstrate the extent to which FMRP targets are associated with risk across a range of psychiatric disorders.

## Materials and methods

### Gene sets

FMRP binding statistics were obtained from Darnell et al. [1], a study of mRNA-FMRP interaction sites in mouse (P11–P25, male) cortical polyribosomes based on crosslinking immunoprecipitation (CLIP) combined with high-throughput RNA sequencing. From 30,999 transcripts, we filtered the data to include only genes detected in the sample (chi-square score > 0), selecting only those (*N* = 8925) for which binding statistics could be obtained. For these genes, we converted Mouse Entrez IDs to human Entrez IDs via their shared HomoloGene ID, obtained from Mouse Genome Informatics (MGI) Vertebrate Homology database release 6.10 (HOM_AllOrganism.rpt, 8th January 2018). Genes that did not convert to a unique protein-coding human homologue (*N* = 330) were excluded. The remaining 8595 genes were ranked by their FMRP binding confidence *P* value and the top 8400 were split into 21 bins of 400 genes which we tested for a relationship between FMRP binding confidence and schizophrenia association. Bin size was selected to balance statistical power with our objective to monitor variance across FMRP binding confidence thresholds.

Functional enrichment analyses were performed using the set of 842 FMRP targets (reported FDR < 0.01 in Darnell et al. [1]) that has been widely used in previous enrichment studies [11, 12].

For comparison, additional FMRP binding statistics were obtained from three recent studies [4, 25, 26]. From a study of hippocampal CA1 pyramidal neurons [25], 10,532 genes were ranked by CLIP score and the top 10,400 split into 26 bins of 400 genes.

Data describing FMRP binding in human frontal cortex were taken from Tran et al. [26]. For each replicate, exonic enhanced CLIP peaks were mapped to genes. Genes were then assigned the *P* value corresponding to that of the most significant peak. In all, 2764 genes identified in both replicates were selected and re-assigned the smallest gene *P* value across replicates. These genes were ranked accordingly and split into 6 bins of 400 and 1 bin of 364 genes.

Lastly, we obtained FMRP binding statistics from human embryonic kidney (HEK) 293 cells [4]. For wild-type FMRP isoforms, exonic binding sites derived from photoactivatable ribonucleoside-enhanced CLIP were mapped to genes. Each gene was assigned the highest PARalyzer peak score from all exonic peaks. Overall, 4736 genes common to both isoforms were selected and re-assigned the smallest gene *P* value across isoforms. Genes were ranked by confidence and divided into 11 bins of 400 genes and 1 bin of 336 genes.

### Samples

#### Common variants

All genetic data were obtained from published case-control studies. Schizophrenia genome-wide association study (GWAS) common variant summary statistics were taken from the Pardiñas et al. [11] study based on a sample of 40,675 cases and 64,643 controls. Bipolar disorder GWAS data were provided by a recent Psychiatric Genomics Consortium (PGC) study [27], consisting of 20,352 cases and 31,358 controls from 32 cohorts of European descent. Major depressive disorder GWAS summary statistics were taken from a PGC meta-analysis of 135,458 cases and 344,901 controls from seven independent cohorts of European ancestry [22]. Alzheimer’s disease GWAS data were obtained from the International Genomics of Alzheimer’s Project “Stage 1” meta-analysis totalling 17,008 case and 37,154 control subjects [28].

#### Rare coding variants

Exome sequencing-derived rare coding variant data from a Swedish schizophrenia case-control study [16] were obtained from the NCBI database of genotypes and phenotypes (dbGaP). After excluding individuals with non-European or Finnish ancestry, and samples with low sequencing coverage, we retained exome sequence in 4079 cases and 5712 controls for analysis.

#### De novo coding variants

De novo mutations were derived [29, 30] from previously published exome sequencing studies of, collectively, 3444 schizophrenia-proband parent trios [12, 30–38] (Supplementary Table 1).

#### Copy number variants (CNVs)

CNV data were compiled from the CLOZUK and Cardiff Cognition in Schizophrenia samples (11,955 cases and 19,089 controls) [39, 40], as well as samples from the International Schizophrenia Consortium (3395 cases and 2185 controls) [41] and the Molecular Genetics of Schizophrenia (2215 cases and 2556 controls) [42], giving a total of 17,565 case and 24,830 control subjects. Genotyping, CNV calling and quality control information can be found in the original reports [39–45].

### Gene set association analysis

Schizophrenia, bipolar disorder, major depressive disorder and Alzheimer’s disease GWAS single nucleotide polymorphisms (SNPs) were filtered to include only those with a minor allele frequency ≥ 0.01. SNP association *P* values were combined (SNP-wise Mean model) into gene-wide *P* values in MAGMA v1.06 [46], using a window of 35 kb upstream and 10 kb downstream of each gene to include proximal regulatory regions. The European panel of the 1000 Genomes Project [47] (phase 3) was used as a reference to account for linkage disequilibrium. Gene sets were tested for enrichment for association with each disorder using one-tailed competitive gene set association analyses in MAGMA, which compares the mean association of genes from the gene set to those not in the gene set, correcting for gene size, linkage disequilibrium and SNP density. The default background was all protein-coding genes.

Case-control exome sequencing data were analysed using Hail (https://github.com/hail-is/hail). We annotated variants using Hail’s Ensembl VEP method (version 86, http://oct2016.archive.ensembl.org/index.html) and defined loss-of-function variants as nonsense, essential splice site and frameshift annotations and nonsynonymous variants as loss-of-function, missense and inframe insertion and deletion mutations. For gene set enrichment tests, we focused on ultra-rare singleton loss-of-function and nonsynonymous variants, that is, those observed once in all case-control sequencing data and absent from the non-psychiatric component of ExAC [48]. Enrichment statistics were generated using a Firth’s penalised-likelihood logistic regression model that corrected for the first ten principal components, exome-wide burden of synonymous variants, sequencing platform and sex.

De novo variant gene set enrichment was evaluated by comparing the observed number of de novo variants in a set of genes to that expected, which was based on the number of trios analysed and per-gene mutations rates [49, 50]. Gene set enrichment statistics for de novo variants were generated by using a two-sample Poisson rate ratio test to compare the enrichment of de novo variants within the gene set to that observed in a background set of genes.

CNV analyses were restricted to CNVs at least 100 kb in size and covered by at least 15 probes. Gene set association was tested by logistic regression, in which CNV case-control status was regressed against the number of set genes overlapped by the CNV, with covariates: CNV size, genes per CNV, study and chip type. To correct for *P* value inflation, empirical *P* values were obtained by calculating the fraction of random size-matched sets of brain-expressed [1] genes that yielded an association as or more significant.

Multiple testing was corrected for using the Bonferroni method.

### Pathway analysis

For Gene Ontology (GO) enrichment analyses, functional annotations of each gene were compiled separately from the GO [51] and MGI Mammalian Phenotype (MP) [52] databases (4 July 2018). GO annotations were filtered to exclude genes with the following evidence codes: NAS (non-traceable author statement), IEA (inferred from electronic annotation), and RCA (inferred from reviewed computational analysis). GO or MP terms containing fewer than 10 genes were then excluded. For all pathway analyses, genes were restricted to those expressed (chi-square score > 0) in the mouse brain tissue used by Darnell et al. [1]. Enrichment of FMRP targets for each GO/MP term was assessed by Fisher’s exact tests, with the contrast group being all remaining expressed genes. Following separate Bonferroni correction for 8270 GO terms or 4606 MP terms, significantly (*P* < 0.01) overrepresented terms were subjected to a competitive refinement procedure to resolve the effects of highly overlapping gene membership between terms. During refinement, terms were re-tested for overrepresentation in FMRP targets following the removal of genes from the term with the highest odds ratio in Fisher’s exact test. Terms that were no longer significant upon re-test (unadjusted *P* > 0.01) were dropped. This was done repeatedly, such that genes from the remaining term with the highest odds ratio on each repeat were removed in addition to those removed on previous iterations.

In primary analyses of genetic association, brain-expressed [1] genes from all overrepresented GO/MP terms (following refinement) were grouped together and divided into those targeted and those not targeted by FMRP, and compared to a background of brain-expressed genes. In secondary analyses, genes from each individual overrepresented term were divided in the same way and tested for association using all protein-coding genes as a comparator. *P* values were Bonferroni corrected for the number of functional terms being tested at each stage of analysis.

We performed a number of tests to investigate the relative enrichments for association between two sets of genes, one a subset of the other. For common variant association, we used the conditional analysis function provided by MAGMA. For rare or de novo coding variants, we compared the effect sizes of the subset of genes with that of the larger set after excluding members of the subset. For the rare coding variant case-control analyses, this was done by performing a *z*-test of beta values, whilst for de novo variant analyses, a two-sample Poisson rate ratio test was used.

In cases where enrichment for genetic association was compared between non-overlapping gene sets, a *z*-test of beta values (common and rare variants) or a two-sample Poisson rate ratio test (de novo variants) was used.

## Results

### The relationship between FMRP binding confidence and enrichment for association with schizophrenia

#### Common variant analysis

**Fig. 1:**
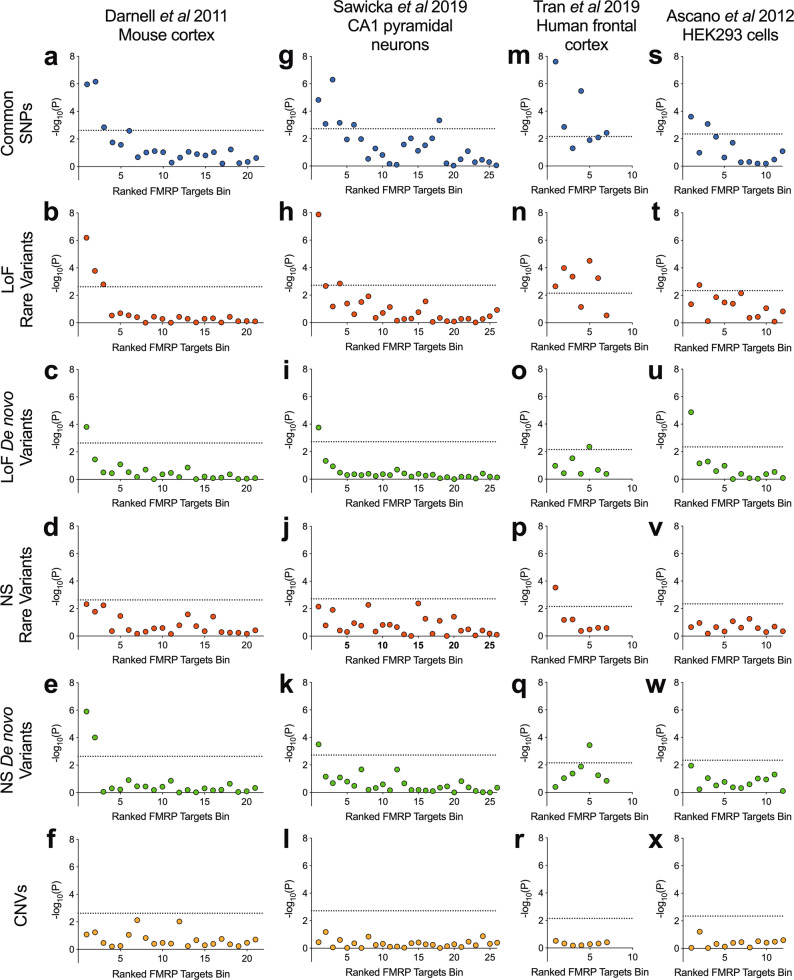
Schizophrenia association of gene sets ranked by FMRP binding confidence in four tissue types. Data of FMRP binding were derived from crosslinking immunoprecipitation studies of mRNA targets in mouse cortex, mouse hippocampal CA1 pyramidal neurons, healthy human frontal cortex and human embryonic kidney (HEK) 293 cells. Genes were ranked by FMRP binding confidence and grouped into bins of 400 genes. Presented are −log_10_(*P*), where the *P* value is derived from gene set association analysis using the genetic variant type shown. CNV analyses were corrected for *P* value inflation using random size-matched sets of expressed genes. Rare coding variants were derived from case-control exome sequencing studies of schizophrenia and defined as variants observed once in all sequenced samples and never in the non-psychiatric component of ExAC. Loss-of-function (LoF) variants include nonsense, splice site and frameshift mutations. Nonsynonymous (NS) variants include loss-of-function and missense variants. Dotted lines indicate a threshold for statistical significance after correction for the number of bins. SNPs single nucleotide polymorphisms, CNVs copy number variants.

We investigated the enrichment for common variant association with schizophrenia in bins of expressed [1] genes (*N* = 400 per bin) grouped by their ranking of mRNA-FMRP binding confidence. These gene set association analyses were performed using MAGMA, in which effects of gene size and SNP density are controlled for within a multiple regression model [46]. Bins containing genes with greater FMRP binding confidence were more enriched for association with schizophrenia (Fig. 1a), with only the top 3 bins being significantly associated (bin 1: corrected *P* = 2.3 × 10^−5^; bin 2: corrected *P* = 1.5 × 10^−5^; bin 3: corrected *P* = 0.030).

#### Rare and de novo coding variant analysis

FMRP targets have likewise been associated with schizophrenia through rare genetic variants [12–17]. We used exome sequencing data to determine which bins of genes were associated with schizophrenia through rare and de novo coding variants. In the case-control analysis of rare variants, notably, the same top three bins enriched for GWAS signal were the only bins to be significantly enriched for association with schizophrenia through rare loss-of-function variants (bin 1: corrected *P* = 1.3 × 10^−5^; bin 2: corrected *P* = 0.0035; bin 3: corrected *P* = 0.034) (Fig. 1b) and only the top bin was significantly enriched for loss-of-function de novo variants (corrected *P* = 0.0033) (Fig. 1c). To examine the contribution from missense and inframe insertion-deletion variants, we repeated the analyses using all nonsynonymous variants. The top two bins were significantly enriched for de novo nonsynonymous variants (bin 1: corrected *P* = 2.65 × 10^−5^; bin 2: corrected *P* = 0.0021) (Fig. 1e). In analyses of rare nonsynonymous variants (Fig. 1d) the topmost bin harboured the most association with schizophrenia but did not exceed the multiple-testing threshold.

#### Copy number variant analysis

Since risk to schizophrenia is also conferred through structural genetic variants [39, 43, 53, 54] in the form of deletions or duplications of large sections of DNA, we investigated whether CNVs from patients with schizophrenia are enriched for genes within bins of probable FMRP targets compared to control subjects. Following logistic regression analysis, no bins surpassed the threshold for significance (Fig. 1f) and the same was true if we examined deletions and duplications separately (Supplementary Fig. 1).

#### Alternative FMRP targets data sets

The mouse cortex-derived FMRP targets [1] analysed above have been the most commonly studied in the psychiatric genetics literature, although FMRP binding statistics from alternative tissues, species and cell populations have been described. We observed that bins containing genes with high FMRP binding confidence in each of mouse hippocampal CA1 pyramidal neurons [25], human frontal cortex [26] and HEK293 cells [4] were significantly enriched for common variant association with schizophrenia (Fig. 1g, m, s) (bin 1, CA1 neurons: corrected *P* = 3.9 × 10^−4^; bin 1, human frontal cortex: corrected *P* = 1.7 × 10^−7^; bin 1, HEK293 cells: corrected *P* = 0.011).

Beyond common variation, the most highly ranked genes for FMRP binding in CA1 pyramidal neurons were similarly enriched for rare loss-of-function variants (bin 1: corrected *P* = 3.5 × 10^−7^), loss-of-function de novo variants (bin 1: corrected *P* = 0.0046) and de novo nonsynonymous variants (bin 1: corrected *P* = 0.0083) (Fig. 1h, i, k).

Gene sets containing high-confidence FMRP targets derived from human cortex samples were enriched for rare variants in cases compared to controls (bin 1, nonsynonymous: corrected *P* = 0.0077; bin 1, loss-of-function: corrected *P* = 0.016) (Fig. 1n, p). However, a lack of low-confidence FMRP target bins, due to the nature of the eCLIP data, limited our ability to draw conclusions concerning the specificity of these associations to the high-confidence binders. The highest ranked bin was not enriched for case de novo variants (bin 1, nonsynonymous: corrected *P* = 1.0; bin 1, loss-of-function: corrected *P* = 0.75) (Fig. 1o, q).

The top ranked gene set defined from HEK293 cells was also associated with schizophrenia through de novo loss-of-function variants (bin 1: corrected *P* = 1.6 × 10^−4^) (Fig. 1u). The same bin was not significantly associated through rare variants; bin 2 was the only gene set to be significantly enriched for rare loss-of-function variants (corrected *P* = 0.021) (Fig. 1t).

Consistent with analyses of gene sets from mouse cortex, bins of genes derived from FMRP binding in the alternative tissues harboured no association with schizophrenia through CNVs (Fig. 1l, r, x).

To summarise, we find that across multiple types of genetic variant and multiple definitions of FMRP targeting, sets of genes more likely to be bound by FMRP harbour greater enrichment for genetic association with schizophrenia.

### Refining schizophrenia association of FMRP targets through functionally defined subgroups

#### Partitioned genetic association by overrepresented functional annotations

Many proteins translated from mRNA targets of FMRP have synaptic functions [1]. In turn, substantial evidence shows that genes encoding proteins with synaptic functions are enriched for genetic association with schizophrenia [11–13, 24, 45, 55]. To further explore the importance of FMRP-dependent translational regulation to the association of genes with schizophrenia, we separated the 842 FMRP target genes, as determined by Darnell et al. [1], into subgroups defined by overrepresented functional categories.

**Fig. 2:**
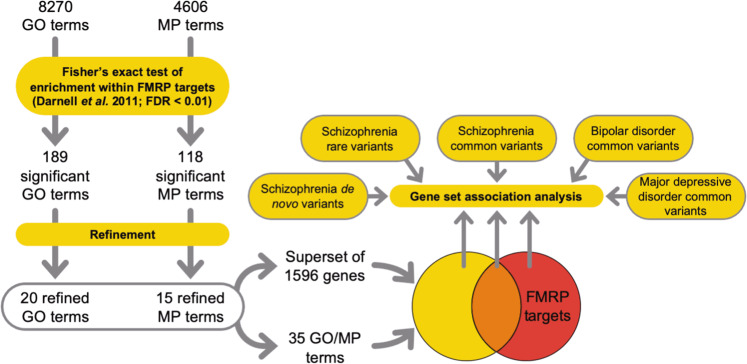
Pathway analysis workflow. Predominant functional subsets of FMRP targets were tested for genetic association with psychiatric disorders. GO gene ontology, MP mammalian phenotype, FDR false discovery rate.

Molecular pathways were derived using pathway analysis (Fig. 2) with GO (Supplementary Table 2) and MP terms (Supplementary Table 3). The resulting 189 GO terms and 118 MP terms were refined to identify terms independently overrepresented among FMRP targets. This procedure left a total of 35 independent overrepresented terms (Supplementary Table 4).

To assess the contribution to genetic association of the property “FMRP binding”, versus that of these functional ontologies, we created a superset (*N* = 1596) of brain-expressed genes, which are included in at least one of the 35 functional terms overrepresented for FMRP targets. FMRP targets from this set (*N* = 401) were strongly enriched for common variant association (*β* = 0.29, corrected *P* = 3.7 × 10^−6^), whilst genes not targeted by FMRP (*N* = 1195) were not (*β* = 0.066, corrected *P* = 0.13) (Table 1). FMRP targets that were not included in any of the 35 terms (*N* = 438) were also significantly associated (*β* = 0.17, corrected *P* = 0.0063). The burden of rare variants and de novo variants in cases showed the same pattern of association, being only enriched in the sets that included FMRP targets (Table 1), regardless of superset membership.

**Table 1:** Partitioning FMRP targets genetic association by overrepresented functional annotation.

Gene set	*N*	Common SNPs		Rare variants		De novo variants	
		*β*	*P*	*β*	*P*	Rate ratio	*P*
Genes exclusive to functional terms	1195	0.066	0.13	LoF: 0.010 NS: 0.015	LoF: 1.0 NS: 1.0	LoF: 0.89 NS: 0.98	LoF: 1.0 NS: 1.0
Overlapping genes	401	0.29	3.7 × 10^−6^	LoF: 0.43 NS: 0.052	LoF: 3.5 × 10^−5^ NS: 0.14	LoF: 1.50 NS: 1.24	LoF: 0.085 NS: 0.014
Genes exclusive to FMRP targets	438	0.17	0.0063	LoF: 0.34 NS: 0.025	LoF: 0.0023 NS: 0.92	LoF: 1.64 NS: 1.35	LoF: 0.024 NS: 1.2 × 10^−4^

GO and MP functional terms independently overrepresented among FMRP targets were merged, then divided by FMRP targets membership. Genes were either exclusively in the functional terms gene set, exclusively in the FMRP targets gene set, or common to both sets (Overlapping genes). Genes not brain-expressed were removed. Background association originating from brain expression was controlled for within gene set association analyses. Shown are the resulting effect sizes (*β* or rate ratio) and *P* values (*P*). For each variant type, *P* values were Bonferroni adjusted for three tests.

*SNPs* single nucleotide polymorphisms, *LoF* loss-of-function, *NS* nonsynonymous.

In direct comparisons of effect sizes from analyses of each type of genetic variant, FMRP targets annotated by overrepresented functional terms were not more enriched for association with schizophrenia than unannotated FMRP targets (common variants: *P* = 0.081; rare loss-of-function variants: *P* = 0.25; rare nonsynonymous variants: *P* = 0.47; de novo nonsynonymous variants: *P* = 0.39; de novo loss-of-function variants: *P* = 0.80). Thus, FMRP targets are enriched for schizophrenia association independently of membership of functional categories (when taken as a whole).

#### Association of FMRP targets from individual GO and MP terms

We next sought to determine from which of the individual overrepresented functional terms FMRP targets capture genetic association with schizophrenia, and whether association is further enriched within FMRP targets from any single overrepresented term, compared to the complete FMRP targets set. Several functionally defined subsets of FMRP targets were significantly associated with schizophrenia through common variation (Table 2), whilst genes not targeted by FMRP were not associated except for those belonging to the term, “calcium ion transmembrane transporter activity” (Supplementary Table 4). However, of the genes in that set, those targeted by FMRP were associated with a significantly greater effect size (*P* = 0.0088) than those not targeted. The “calcium ion transmembrane transporter activity” fraction of FMRP targets (*N* = 25) remained significantly associated with schizophrenia even after conditioning on all FMRP targets (Supplementary Table 4), implying that this functionally defined subset of FMRP targets is more enriched for association with schizophrenia than FMRP targets as a whole. No other term captured FMRP targets with a significantly greater enrichment of genetic association than the full FMRP targets gene set.

**Table 2:** GO and MP terms overrepresented among FMRP targets derived from mouse cortex which capture a significant (*P*.adj < 0.05) portion of the common variant genetic association with schizophrenia.

Term	Genes not FMRP targets				Genes FMRP targets			
	*N*	*β*	*P*	*P*.adj	*N*	*β*	*P*	*P*.adj
Calcium ion transmembrane transporter activity (GO: 0015085)	91	0.419	4.7 × 10^−4^	**0.017**	25	1.080	6.9 × 10^−6^	**2.4** **×** **10**^**−4**^
Abnormal motor coordination/balance (MP: 0001516)	538	0.104	0.028	0.97	117	0.463	2.8 × 10^−5^	**9.6** **×** **10**^**−4**^
Abnormal seizure response to inducing agent (MP: 0009357)	125	0.190	0.043	1.0	42	0.710	1.1 × 10^−4^	**0.0038**
Abnormal spatial learning (MP: 0001463)	141	0.161	0.057	1.0	61	0.569	1.4 × 10^−4^	**0.0049**
Growth cone (GO: 0030426)	50	0.245	0.077	1.0	27	0.854	1.7 × 10^−4^	**0.0060**
Abnormal nest building behaviour (MP: 0001447)	15	0.265	0.22	1.0	12	1.290	2.0 × 10^−4^	**0.0071**
Abnormal excitatory postsynaptic currents (MP: 0002910)	60	0.177	0.12	1.0	35	0.715	3.5 × 10^−4^	**0.012**
Axon part (GO: 0033267)	108	0.134	0.12	1.0	54	0.505	6.6 × 10^−4^	**0.023**

Shown are effect sizes (*β*) and *P* values (*P*) in gene set association analysis of genes targeted, or not targeted, by FMRP. *P* values were Bonferroni adjusted (*P*.adj) for 35 terms. *P* values statistically significant after adjustment are shown in bold.

Rare loss-of-function variants from patients with schizophrenia were enriched in FMRP targets from two terms (abnormal spatial learning and abnormal motor coordination/balance) (Supplementary Table 5), whilst no association was found between rare coding variants in non-targeted genes from each term and schizophrenia. None of these subsets harboured significantly more enrichment for case variants than all FMRP targets.

None of the subsets tested captured a significant burden of case de novo nonsynonymous variants. Conversely, enrichment for de novo loss-of-function variants was observed for FMRP targets of two terms (learning and abnormal spatial learning) (Supplementary Table 6), which was not reflected by any subsets not targeted by FMRP.

Overall, these analyses suggest that the overrepresentation of FMRP targets is the property that best captures genetic association of these biological pathways with schizophrenia, not the biological pathway itself.

### Genetic association of FMRP targets in other psychiatric disorders

**Fig. 3:**
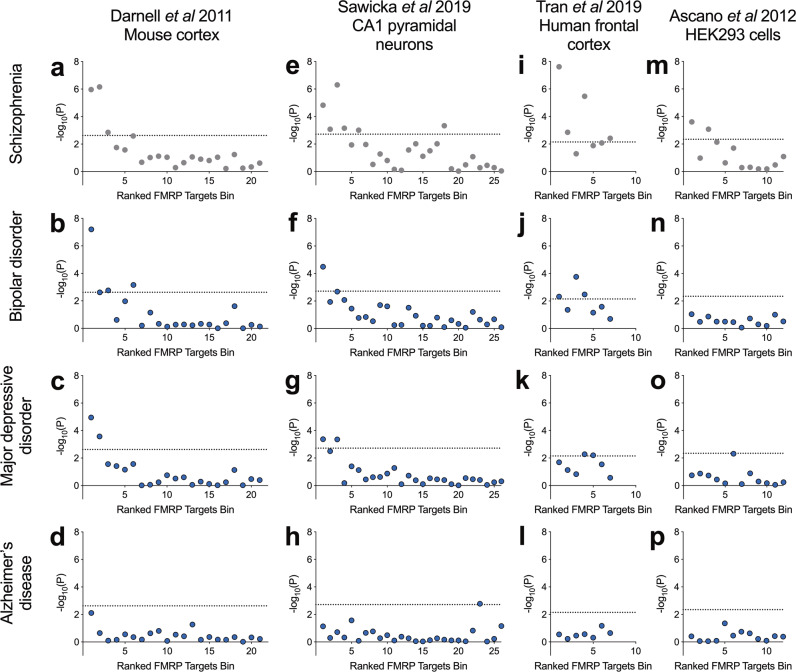
Common variant association of FMRP target bins with schizophrenia, bipolar disorder, major depressive disorder and Alzheimer’s disease. Data of FMRP binding in four tissue types were derived from the sources shown. Presented is −log_10_(*P* value) following common variant gene set association analysis of bins of 400 genes ranked by FMRP binding confidence. Dotted lines represent a threshold for statistical significance after correction for the number of bins. Data presented in the top row of panels (**a**, **e**, **i**, **m**) are duplicated from Fig. 1.

Schizophrenia shares substantial genetic susceptibility with bipolar disorder and major depressive disorder [56–59] and FMRP targets have been previously associated through common variation with major depressive disorder [22]. For comparison across disorders, we tested the enrichment of FMRP targets bins for association with major depressive disorder and bipolar disorder using common variant data from GWAS. In both sets of analyses, there was a clear relationship between mouse cortex FMRP binding confidence and genetic association (Fig. 3b, c). The topmost bin, containing genes most likely to be FMRP targets, was the most strongly enriched for association with bipolar disorder (corrected *P* = 1.4 × 10^−6^) and major depressive disorder (corrected *P* = 2.5 × 10^−4^). Conversely, no bins were significantly enriched for association with Alzheimer’s disease (Fig. 3d), implying a degree of specificity to the association of high-confidence FMRP targets to psychiatric versus neurodegenerative disorders.

These analyses were repeated for bins of FMRP targets derived from other sources. Consistent with our previous observations, we found that the most highly ranked genes from FMRP binding in mouse CA1 pyramidal neurons were also the most strongly enriched for association with bipolar disorder (corrected *P* = 8.3 × 10^−4^) and major depressive disorder (corrected *P* = 0.011) (Fig. 3f, g). Highly ranked bins of FMRP targets defined in human frontal cortex showed association with bipolar disorder (bin 1: corrected *P* = 0.034) but less with major depressive disorder (bin 1: corrected *P* = 0.14) (Fig. 3j, k). No bins derived from binding in HEK293 cells surpassed significance for association with bipolar disorder or major depressive disorder (Fig. 3n, o). Genetic association with Alzheimer’s disease was not enriched in highly ranked gene sets from any tissue type.

We investigated functionally annotated subgroups of FMRP targets for association with bipolar disorder and major depressive disorder. Beyond background association from brain-expressed genes, FMRP targets from mouse cortex annotated for membership of the 35 overrepresented pathways were strongly associated with bipolar disorder (*β* = 0.23, corrected *P* = 1.6 × 10^−5^) and major depressive disorder (*β* = 0.21, corrected *P* = 1.6 × 10^−5^), whilst genes from the same functional terms not targeted by FMRP harboured no significant association (bipolar disorder: *β* = 0.037, corrected *P* = 0.38; major depressive disorder: *β* = 0.031, corrected *P* = 0.49) (Table 3). A similar picture was observed for individual overrepresented GO/MP terms. FMRP targets from four terms (calcium ion transmembrane transporter activity, abnormal nest building behaviour, abnormal spatial learning and abnormal seizure response to inducing agent) were significantly associated with bipolar disorder. Notably, genetic association of FMRP targets from these four terms was common to schizophrenia and bipolar disorder. FMRP targets from one term (abnormal synaptic vesicle morphology) were significantly associated with major depressive disorder (Supplementary Table 4). FMRP targets belonging to the term abnormal nest building behaviour (*N* = 12) were more highly enriched for association with bipolar disorder than FMRP targets as a whole. No subsets of FMRP targets were significantly more enriched for association with major depressive disorder than the full FMRP targets set (Supplementary Table 4).

**Table 3:** Partitioning FMRP targets common variant association by overrepresented functional annotation.

Gene set		Schizophrenia		Bipolar disorder		Major depressive disorder	
	*N*	*β*	*P*	*β*	*P*	*β*	*P*
Genes exclusive to functional terms	1195	0.066	0.13	0.037	0.38	0.031	0.49
Overlapping genes	401	0.29	3.7 × 10^−6^	0.23	1.6 × 10^−5^	0.21	9.7 × 10^−5^
Genes exclusive to FMRP targets	438	0.17	0.0063	0.14	0.0074	0.15	0.0026

Gene sets were formed from genes exclusive to the functional terms gene set, genes exclusive to the FMRP targets gene set, or genes common to both sets (Overlapping genes). Gene set association analyses were performed using a background of brain-expressed genes to account for background association. Shown are the effect sizes (*β*) and *P* values (*P*) from gene set association analyses using MAGMA. For each disorder, *P* values were adjusted for three genes sets using the Bonferroni method. Data for schizophrenia are repeated from Table 1.

## Discussion

In this study we show that genes with high probability of being targets of FMRP are enriched for association with schizophrenia, bipolar disorder and major depressive disorder. We also show that it is the property of being an FMRP target that better captures the genetic association, rather than membership of other gene sets that are both associated with schizophrenia and enriched for targets of FMRP.

Only bins of genes with high FMRP binding confidence were enriched for association with schizophrenia through common variation, exome sequencing-derived rare variation and exome sequencing-derived de novo rare variation. This same relationship was reflected across multiple FMRP binding data sets, and in analyses of bipolar disorder and major depressive disorder. Our observations are consistent with previous gene set analyses of FMRP targets in the context of schizophrenia [11–15] and major depressive disorder [22], but whilst FMRP targets have been previously linked to bipolar disorder through rare coding variants [60], our findings provide novel evidence linking FMRP targets to bipolar disorder through common variation.

Despite the evidence implicating FMRP targets in psychiatric disorders [11–15], the overrepresentation of long, brain-expressed genes with synaptic functions has led to concerns over the validity of the link to FMRP [23]. It is therefore important to stress that the methods used here, and previously [11], correct for, or are unaffected by, gene length. Furthermore, whilst association was derived from expressed mRNAs in mouse brain, it did not generalise to bins of brain-expressed genes with low FMRP binding confidence.

Consistent with previous pathway analysis [1], we note that a substantial proportion of FMRP targets have functions related to synaptic activity, anatomy or development. FMRP activity is regulated in response to neuronal activity [61–64], and is an important mediator of synapse development [65–67], synaptic plasticity [68–70], learning and memory [71–73]. Genetic and functional studies have highlighted the relevance of perturbed synaptic plasticity in psychiatric disorders [12, 27, 44, 45, 74–76], although we find that the risk conferred by variants affecting such pathways overrepresented among FMRP targets is concentrated within the fraction of genes targeted by FMRP. Hence, despite the convergence of psychiatric risk on synaptic pathways [12, 24, 27, 75–77], the association of FMRP targets was not attributed to these overrepresented annotations. Instead, it appears that there is a degree of specificity to this risk, such that genes regulated locally by FMRP during activity-induced synaptic plasticity, required for development or learning, are most relevant to psychiatric disorder. Whilst it could be argued that a further, larger set of genes could account for the enrichment of genetic association observed in FMRP targets, a recent schizophrenia GWAS [11] emphasised the independence of the association, notably as the only gene set that was associated independently of genes defined by loss-of-function intolerance.

It should be noted that other synapse-related gene sets are enriched for association with psychiatric disorders independently of FMRP targets [11]. Here we found that, whilst strongest for genes targeted by FMRP, genes involved in “calcium ion transmembrane transporter activity” held independent association with schizophrenia. However, the strongly associated, albeit small, intersection between genes from this set and FMRP targets contained a stronger enrichment of schizophrenia common variant association than FMRP targets (or indeed the GO term) as a whole. This is consistent with previous evidence for association of calcium channels with schizophrenia [10, 11, 13], yet additionally suggests that FMRP captures a subset of genes related to calcium ion transport in which common variant association is concentrated. The regulation of calcium channel activity by FMRP may be an important area of continued investigation, in the context of schizophrenia.

We demonstrate that high-confidence FMRP target gene sets derived from multiple sources are associated with schizophrenia and other psychiatric disorders. Beyond the commonly studied gene set from mouse cortex [1], highly ranked genes derived from studies of FMRP binding in human frontal cortex [26], mouse hippocampal CA1 pyramidal neurons [25] and HEK293 cells shared the enrichment for genetic association, albeit with differing profiles of association across types of genetic variant. Our observations from mouse cortical tissue were, perhaps unsurprisingly, most consistently mirrored by mouse pyramidal neurons, whilst gene sets formed from FMRP binding statistics in human cortex and HEK293 cells conferred risk to schizophrenia through different combinations of mutation type. Furthermore, in comparisons across psychiatric disorders, high-confidence FMRP targets in HEK293 cells were enriched for common variant association with schizophrenia, but not bipolar disorder or major depressive disorder. However, it is notable that variability in the number of genes for which FMRP binding statistics were available may influence our findings. Differences in the study design and methods employed by the research groups could modify the capture or ranking of mRNA targets. Additional investigation of FMRP target conservation across species, tissues and cell types using a unified methodology will facilitate the partitioning of genetic association among them.

High-confidence FMRP targets were not enriched in CNVs from schizophrenia cases when compared to CNVs from controls. Whilst FMRP targets have been consistently implicated in schizophrenia from analyses across other types of genetic variant, studies of structural variation in schizophrenia have shown only modest association for FMRP targets [45, 54, 78]. However, deletions at the 15q11.2 locus encompassing the FMRP interactor, CYFIP1—required for FMRP-dependent translational regulation [5, 79]—are associated. Notably, case CNVs have been shown to be enriched for components of synaptic signalling complexes [45, 55], which, together with the current findings, suggests that not all synaptic signalling pathways associated with schizophrenia are under the regulation of FMRP.

Our observations resonate with the growing body of literature challenging the biological validity of viewing major psychiatric disorders as discrete entities with independent genetic aetiology [80–83]. There is considerable overlap between the genetic risk attributable to schizophrenia, bipolar disorder and major depressive disorder [56–59]. The present (and published) findings highlight that FMRP targets are a point of biological convergence. Additional evidence suggests that genetic association of FMRP targets may extend also to autism [18–21] and attention-deficit hyperactivity disorder [84].

Our findings highlight a set of genes regulated through a common mechanism that harbour risk across several psychiatric disorders. However, there is still a degree of uncertainty as to precisely which mRNAs are regulated by FMRP. Multiple studies have examined this, each yielding overlapping, yet distinct sets of FMRP targets [1, 4, 25, 26, 85–88]; some of the variability likely originating from tissue specificity. When performing pathway analyses with genomic data, many studies, including this one, have obtained FMRP targets from an investigation of mRNA-FMRP interaction sites in mouse cortical polyribosomes [1], in which membership was assigned by applying a stringent cut-off to a continuous scale of binding confidence, likely resulting in some false positives and more false negatives. Moreover, binding by FMRP may not equate to translational repression in the cell, which requires additional contribution from binding partners CYFIP1 and eIF4E, within a protein complex [5]. As well as regulating protein synthesis, FMRP is reported to have other roles, including the mediation of mRNA stability and dendritic transport [9, 89, 90]. Each of these processes may be relevant to psychiatric risk and this line of research will benefit from further validation of FMRP-regulated mRNAs.

Whilst highly heritable, the emergence of schizophrenia, bipolar disorder and major depressive disorder is in most cases attributable to joint effects of genetic and environmental risk factors. Likewise, environmental factors influence cognitive and behavioural deficits in people with fragile X syndrome [91, 92]. Consistent with genetic observations, a convergence on synaptic dysfunction is also described in studies of non-genetic and epigenetic factors contributing to psychiatric disorders [93]. However, the interaction between FMRP function and environmental exposures in the context of psychiatric disorders has not been studied. Continued investigation of liability to psychiatric disorders deriving from FMRP targets could benefit from examining these relationships, both across neurodevelopment and during adult plasticity.

Our results serve to strengthen the evidence that a population of genes targeted by FMRP, many of which have synaptic functions, are affected by genetic variation conferring risk to psychiatric disorders, including schizophrenia, bipolar disorder and major depressive disorder. We conclude that targeting by FMRP is currently the most suitable functional annotation to reflect the origin of these associations and represents a common mode of regulation for a set of genes contributing risk across several major psychiatric presentations.

## Supplementary information

Supplementary Information

Supplementary Tables
